# Editorial: Professionalization in adaptive and Paralympic sports: current trends and future directions

**DOI:** 10.3389/fspor.2026.2006106

**Published:** 2026-10-12

**Authors:** Ulrich Theobald, Christine Maleske, Eric M. Lamberg

**Affiliations:** 1 Faculty for Special Education, Ludwigsburg University of Education, Ludwigsburg, Germany; 2 Sport Management, School of Kinesiology, University of Michigan, Ann Arbor, MI, United States; 3 School of Health Professions, Stony Brook University, Stony Brook, NY, United States

**Keywords:** adaptive sports, disability, elite-level athletes, Paralympic sport, parasports, professionalization, sport policy

The field of adaptive and Paralympic sports has experienced a substantial increase in visibility, popularity, and societal recognition over recent decades. Major events such as the Seoul 1988 Summer Paralympic Games and, more prominently, the Paris 2024 Paralympics have established new benchmarks in terms of media coverage, infrastructural development, and public engagement. These developments have been accompanied by significant increases in both public attention and financial investment.

In this editorial, adaptive sport refers to sport opportunities designed or modified to facilitate participation by individuals with disabilities, whereas Paralympic sport refers specifically to sports governed within the Paralympic Movement and competition structure. This rising prominence has generated a corresponding need for greater professionalization across all areas of these sports, inviting comparisons with established and developing high-performance sport systems. The implications of increasing professional standards are multifaceted, affecting athlete development and performance diagnostics, coaching practices, organizational structures, and community and governmental support mechanisms. Consequently, there is a growing demand for differentiated and in-depth research that draws on diverse disciplinary perspectives and methodological approaches.

With this in mind, the objective of the present editorial is to highlight and analyze professionalization processes in adaptive and Paralympic sports – evaluating how these processes influence the sports involved, their corresponding communities as well as public policies. By identifying ongoing dynamics and future trajectories, this contribution seeks to provide insight and to advance scholarly discussion on the challenges and opportunities associated with the continued evolution and maturation of the field. In doing so, it adopts a comprehensive perspective on relevant systems and structures, as well as the various factors that influence them.

To illustrate how these processes of professionalization are currently being examined across the field, the submitted manuscripts can be categorized into two overarching domains: (1) contributions addressing elite sport and (2) those concerned with public and organizational policy. In the context of elite-level performance in adaptive and Paralympic sport, Carroll et al. contributed two papers addressing how to effectively identify and develop talented athletes. The first is a scoping literature review that outlines the current state of research, while the second is a Delphi study involving surveys with experts in the field.

Two contributions highlight aspects directly linked to high-performance training and coaching in Paralympic sports: Øvstehage et al. conducted a qualitative study with current and former Norwegian elite-level athletes from different disciplines with the aim to identify prerequisites for training quality. Theobald et al. conducted semi-structured interviews with coaches from a Paralympic Training Center in Germany in order to outline different facets of individual professionalization.

Several manuscripts are related to research questions about physical and psychosocial health and well-being of adaptive and Paralympic athletes. Winckler et al. undertook a literature review on the effects that scientific advances and conceptual transformations have on health care, the classification system, training, and competition in Paralympic sports. This was done with the goal to highlight the impact of major sport events on the development of assistive technologies. Lee et al. explored the impact of participation in amputee soccer on biopsychosocial functioning among athletes using surveys and semi-structured interviews with active members of the American Amputee Soccer Association in the United States. Garcia-Carillo et al. did a single-case study with an elite-level Paralympic shot-putter in order to investigate physical and mental effects of long-haul travel that can be linked to performance in competition.

Biomechanical research data is presented in two further works. Garcia-Carillo et al. give an overview of the current literature on the science of adapted throws in multiple events including shot put, discus, javelin, and club. In their systematic review they investigated scientific evidence guiding technique, training and classification, summarizing studies from biomechanics, physiology and psychosocial domains. In their contribution on golf swing performance, Jones et al. examine launch data of golf shots that was collected in a field test to give an overview of performance variables in golfers with disabilities.

The other main category of manuscripts are papers that deal with research questions concerning public and organizational policies in the field of adaptive and Paralympic Sports. Revuelta and Hernández comparatively analyze public policies for the promotion of Paralympic sport in ten Latin American countries. Their study is based on a comparative documentary analysis. Using quantitative surveys, Ruiz et al. explore determining factors for participation in Paralympic sports programs in Latin America. Organizational policies are examined by Arthur-Banning et al. drawing on qualitative methods and interview data from members of the International Federation of Cerebral Palsy Football, they identify barriers to and facilitators of participation in international cerebral palsy football events. Pierre-Glaude et al. examined barriers and facilitators associated with developing and delivering a community-based adaptive sports program for youth with limb loss and limb difference.

While all manuscripts align closely with the scope of the editorial, they offer diverse perspectives and are characterized by considerable methodological variation. Collectively, they can be grouped into several broad methodological approaches. A first group comprises literature-based studies, including the scoping review by Carroll et al., as well as the narrative and systematic reviews conducted by Winckler et al. and Garcia-Carillo et al., respectively. These contributions primarily aim to synthesize existing knowledge, with the Delphi approach additionally incorporating structured expert consensus.

A second group encompasses studies that use qualitative research designs. For example, Øvstehage et al. use a hermeneutic phenomenological approach, while Arthur-Banning et al., Theobald et al., and Lee et al. employ qualitative semi-structured interviews. Lee et al. further combine these interviews with survey data, thereby bridging qualitative and mixed-methods approaches. The Delphi study by Carroll et al. also falls within this group.

A third category includes quantitative designs. Ruiz et al. utilize quantitative survey data, and Jones et al. analyze performance-related field data, representing an applied, measurement-oriented approach within sport science research.

Finally, further methodological perspectives are reflected in more specialized designs, such as the single-case study by Garcia-Carillo et al. and the comparative documentary analysis conducted by Revuelta and Hernández, both of which provide context-specific and analytically focused insights.

The contributions assembled in this Research Topic collectively underscore the growing complexity and professionalization of adaptive and Paralympic sports. They reveal key insights into talent identification, high-performance training, organizational policy, and athlete well-being, while also highlighting significant gaps that warrant further scholarly attention. Based on the current Research Topic, several promising avenues for future research emerge.

First, there is a clear need for expanded examination and cross-comparison of public and organizational policies across regions worldwide. While the present issue includes valuable analyses of policy frameworks in Latin America, comparable investigations in other world regions—particularly Africa and Asia—are notably absent. Such comparative work could enable a more comprehensive understanding of how differing political, economic, and cultural contexts shape the development and professionalization of adaptive and Paralympic sport systems.

Second, future research should investigate how adaptive athletes and Paralympians transition into professional sport, with a particular emphasis on their career journeys and factors that promote or hinder their ability to become professional athletes. Longitudinal studies tracking athletes from grassroots participation through elite and professional levels could illuminate critical support structures, barriers, and decision points along these pathways.

Building on such athlete-centered pathway research, there is also a need to explore how (a) coaches and (b) general staff and administrators—working in professional adaptive sport leagues and teams and/or within National Paralympic Committees (NPCs) and the International Paralympic Committee (IPC)—first became involved in professionalized adaptive and Paralympic sports. Understanding the recruitment, training, and professional development trajectories of these key stakeholders would provide valuable insights into the human infrastructure underpinning high-performance adaptive sport systems.

Although this issue includes manuscripts on biomechanics, high-performance training, and coaching in Paralympic sports, additional work is needed to better capture the distinctiveness and training needs of adaptive and Paralympic athletes. Current coverage spans only a limited range of sports and there is a need for evidence-based approaches to support coaches in developing athlete performance toward professionalization across all adaptive and Paralympic disciplines. Future studies should aim to develop sport-specific and training models that account for the unique physiological, psychosocial, technical, and tactical demands faced by athletes across the full spectrum of adaptive sports.

While the manuscripts in this Research Topic focus on current trends and the future of professional adaptive and Paralympic sports, it may also be valuable to examine the effects of pursuing professional athletic pathways on adaptive athletes and Paralympians—specifically physical effects, psychosocial effects, and overall impacts on well-being. Research in this area could inform the design of holistic support systems that address not only performance optimization but also long-term health, career sustainability, and quality of life for athletes navigating professional careers.

In addition, as adaptive sport continues to professionalize alongside disciplines represented within the Paralympic Games, it will become increasingly important to examine how organizations respond to changing patterns of consumption, fandom, media coverage, sponsorship and stakeholder engagement. It will be important to understand how organizations adapt, change, and/or react to any trends related to the above. Studies exploring organizational responses to shifting audience expectations, commercial pressures, and media dynamics would contribute to a more nuanced understanding of the evolving ecosystem of professional adaptive and Paralympic sports.

Taken together, these directions point to a rich and multifaceted research agenda. By addressing these gaps, future academic work can help ensure that the professionalization of adaptive and Paralympic sports proceeds in ways that are equitable, sustainable, and supportive of all stakeholders involved.

